# Intima-media thickness cut-off values depicting “halo sign” and potential confounder analysis for the best diagnosis of large vessel giant cell arteritis by ultrasonography

**DOI:** 10.3389/fmed.2022.1055524

**Published:** 2022-12-13

**Authors:** Marcin Milchert, Jacek Fliciński, Marek Brzosko

**Affiliations:** Department of Internal Medicine, Rheumatology, Diabetology, Geriatrics and Clinical Immunology, Pomeranian Medical University, Szczecin, Poland

**Keywords:** giant cell arteritis, arteriosclerosis, vasculitis, ultrasound, halo sign, reference range

## Abstract

**Background:**

Vascular ultrasound enables fast-track diagnosis of giant cell arteritis (GCA), but this method remains subjective. We aimed to determine intima-media thickness (IMT) cut-off values for large vessel GCA (LV-GCA) and identify the clinically relevant factors influencing it.

**Methods:**

We included 214 patients referred for ultrasound evaluation within a fast-track clinic due to suspected GCA. IMT was measured in axillary, brachial, subclavian, superficial femoral, and common carotid arteries (CCA), in a place without identifiable atherosclerotic plaques. IMT cut-off values for vasculitis were determined by comparing measurements in arteries classified as vasculitis vs. controls without GCA/polymyalgia rheumatica (PMR).

**Results:**

Giant cell arteritis was diagnosed in 81 individuals, including extracranial LV-GCA in 43 individuals. Isolated PMR was diagnosed in 50 subjects. In 83 remaining patients, another diagnosis was confirmed, and they served as controls. The rounded optimal IMT cut-off values for the diagnosis of axillary vasculitis were 0.8 mm, subclavian-0.7 mm, superficial femoral-0.9 mm, CCA-0.7 mm, and brachial-0.5 mm. The IMT cut-off values providing 100% specificity for vasculitis (although with reduced sensitivity) were obtained with axillary IMT 1.06 mm, subclavian-1.35 mm, superficial femoral-1.55 mm, CCA-1.27 mm, and brachial-0.96 mm. Axillary and subclavian arteritis provided the best AUC for the diagnosis of GCA, while carotid and axillary were most commonly involved (24 and 23 patients, respectively). The presence of calcified atherosclerotic plaques was related to an increase of IMT in both patients and controls, while male sex, age ≥ 68, hypertension, and smoking increased IMT in controls but not in patients with GCA.

**Conclusion:**

Cut-off values for LV-GCA performed best in axillary and subclavian arteritis but expanding examination to the other arteries may add to the sensitivity of GCA diagnosis (another location, e.g., brachial arteritis) and its specificity (identification of calcified atherosclerotic plaques in other arteries such as CCA, which may suggest applying higher IMT cut-off values). We proposed a more linear approach to cut-off values with two values: one for the most accurate and the other for a highly specific diagnosis and also considering some cardiovascular risk factors.

## Key messages

- Intima-media cut-off values for large vessel GCA perform best in axillary and subclavian arteritis.
- Expanding examination to multiple arteries adds to the diagnostic sensitivity and specificity.
- The presence of some cardiovascular risk factors (male sex, age ≥ 68, hypertension, smoking, and the presence of calcified atherosclerotic plaques) may confound intima-media cut-off values.
- We proposed prior confounder analysis to apply cut-off values with two values: one for most accurate and the other for highly specific diagnosis.

## Introduction

**Figure 1:**
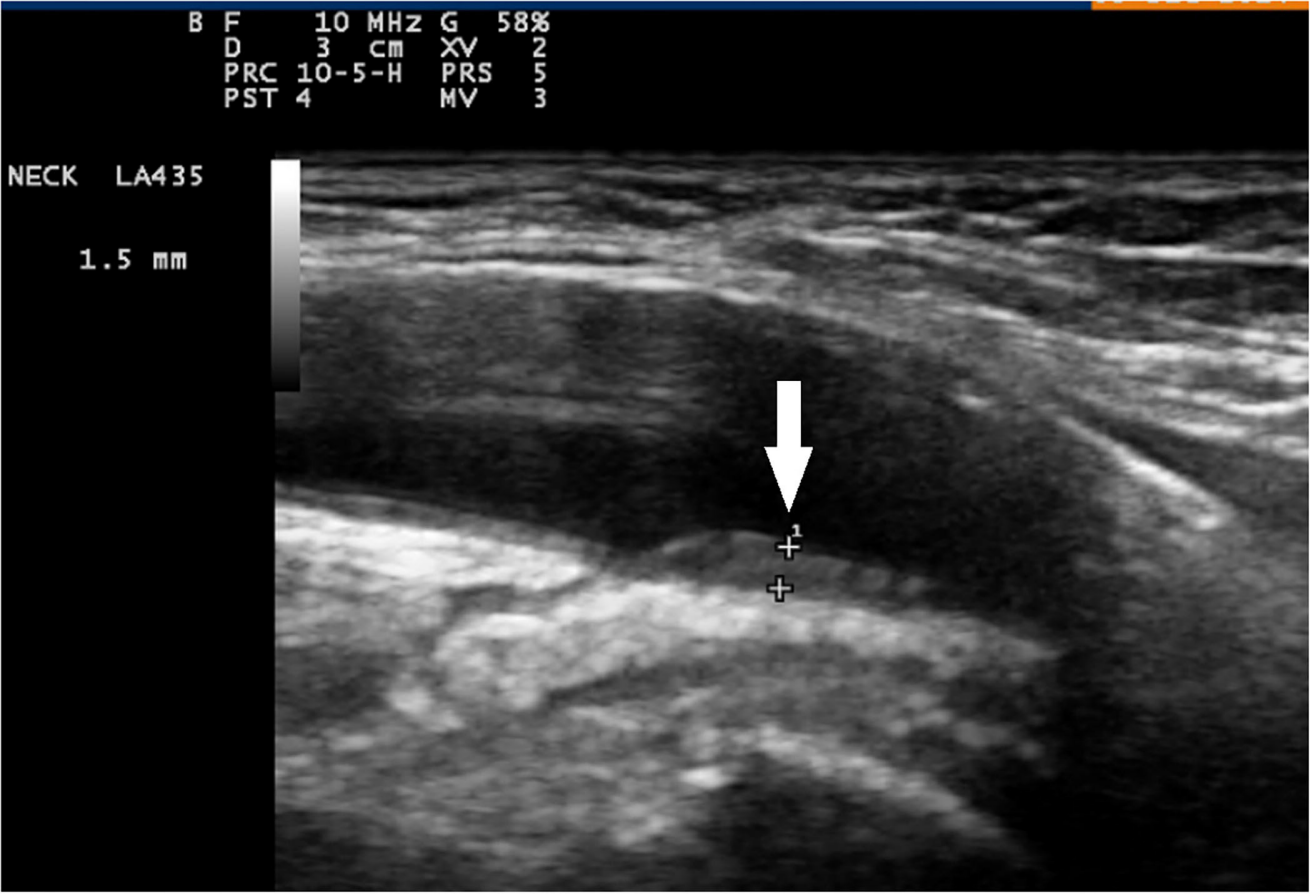
Color duplex sonography of axillary artery and longitudinal plane. Signs of vasculitis in an axillary artery (arrow) sliding down to a normal intima-media in the brachial artery (slope sign).

Diagnosis of giant cell arteritis (GCA) with large vessel involvement is delayed compared to temporal arteritis but is improving, thanks to the wider implementation of imaging in clinical practice (1, 2). Ultrasound-based fast-track clinics enable fast diagnosis of GCA and rapid initiation of treatment to prevent disease complications (3). Ultrasonography is a promising method for the diagnosis of not only temporal arteritis (4) but also extracranial, large vessel GCA (LV-GCA) (5–9). Compared to temporal artery biopsy, color duplex ultrasonography (CDU) offers faster results and is non-invasive and cost-effective, while serving high sensitivity and specificity (10). The most important ultrasonographic sign of large artery wall inflammation is an increase of intima-media thickness (IMT) with some characteristic features known as the “halo sign” (Figure 1) (11, 12). However, in elderly patients with GCA, atherosclerosis is common and requires careful differentiation with an increase of IMT caused by vasculitis (13, 14). Observer dependency and lack of standardization of the ultrasound method remain major concerns. Modern high-frequency ultrasound probes provide resolutions that enable exact vessel wall thickness measurements. There is a need to define IMT consistent with the halo sign for the diagnosis of GCA and identify the potential factors that influence IMT. Therefore, we aimed to determine IMT cut-off values for LV-GCA in a real-life scenario considering patients' cardiovascular risk factors as potential confounders.

## Materials and methods

### Study population

Between April 2011 and June 2015, 312 patients suspected of GCA were referred for CDU evaluation within a fast-track GCA clinic. Referrals for GCA evaluation were not limited, and they included ophthalmological manifestations, other manifestations of cranial GCA, polymyalgia rheumatica (PMR) manifestations, pyrexia of unknown origin, and others. All consecutive, confirmed GCA cases were included but 98 controls (negative temporal and large vessel CDU and low clinical probability of GCA) were not included due to the loss of follow-up. They were not referred back for GCA reevaluation although our department is the only local reference center for vasculitis. We included isolated PMR as a distinct subgroup in the analysis to evaluate possible atherosclerosis in this subgroup and its risk factors. We finally included 214 subjects in the study. A history of hypertension, smoking, and diabetes mellitus was assessed. Arterial hypertension was defined as systolic blood pressure of ≥140 mm Hg and/or diastolic blood pressure of ≥90 mm Hg. Diabetes mellitus was defined in accordance with the Polish Society of Diabetology guidelines as a positive oral glucose tolerance test, a random glucose level of ≥200 mg/dl with clinical manifestations, or with fasting glucose of ≥126 mg/dl. Hypercholesterolemia was defined as an LDL cholesterol level of ≥115 mg/dl. Smoking was noted in case of a history of >3 pack years of smoking. Fasting lipid profiles were measured or retrieved from the medical records (within 1 year prior to GCA diagnosis) in 137 patients. Temporal artery biopsy (TAB) was performed in 44 patients, and contrasted computed tomography (CT) of the thoracic and abdominal aorta was performed in 126 patients. The research was approved by the decision KB-0012/111/10 and KB-0012/12/14 of the Local Ethical Committee.

### GCA diagnosis

Final GCA and PMR diagnoses were established by a team of a minimum of two experienced rheumatologists and confirmed in at least one follow-up visit within 9 months. Patients with cranial GCA met ACR criteria (15). Large vessel GCA diagnosis was confirmed by arterial ultrasound and/or aortic CT (defined as circumferential, long-segmental aortic wall thickening of ≥3 or ≥2 mm with adjacent adventitia involvement) together with the presence of GCA or PMR manifestations. Patients with PMR met the 2012 EULAR/ACR classification criteria (16).

### Ultrasound examination

All CDU, together with categorization into arteritis and non-arteritis findings, were performed before or within 3 days after treatment initiation, by a single physician (M.M.), experienced with performing >800 ultrasound examinations in suspected LV-GCA. He was blinded to diagnosis but aware of clinical presentation. Bilateral CDU examination of brachial, axillary, subclavian, superficial femoral, and common carotid arteries (CCA) was performed in all patients in both transverse and longitudinal planes. Halo sign definition was consistent with the one formulated by OMERACT: homogenous, hypoechoic wall thickening, well-delineated toward the luminal side, visible both in longitudinal and transverse planes, and most commonly concentric in transverse scans (11). Maximal IMT was measured from vessel lumen to media-adventitia interface, in mm with two decimals, in a place without identifiable atherosclerotic plaques, preferably on the distal wall. Maximal IMT from the left and right side locations were noted as well as mean IMT of corresponding left and right side measurements were calculated, which has to be included in further analysis. IMT cut-off values for vasculitis were determined by comparing measurements in arteries classified as vasculitis vs. controls. Additionally, measurements in arteries classified as vasculitis were compared with corresponding locations in GCA without large vessel vasculitis. The presence of calcified atherosclerotic plaques covering over 25% of vessel lumen in carotid bulbs and femoral arteries was assessed with CDU and noted. Esaote MyLab25Gold machine with 5–10 and 10–18 MHz linear probes was used.

### Statistical analysis

**Figure 2:**
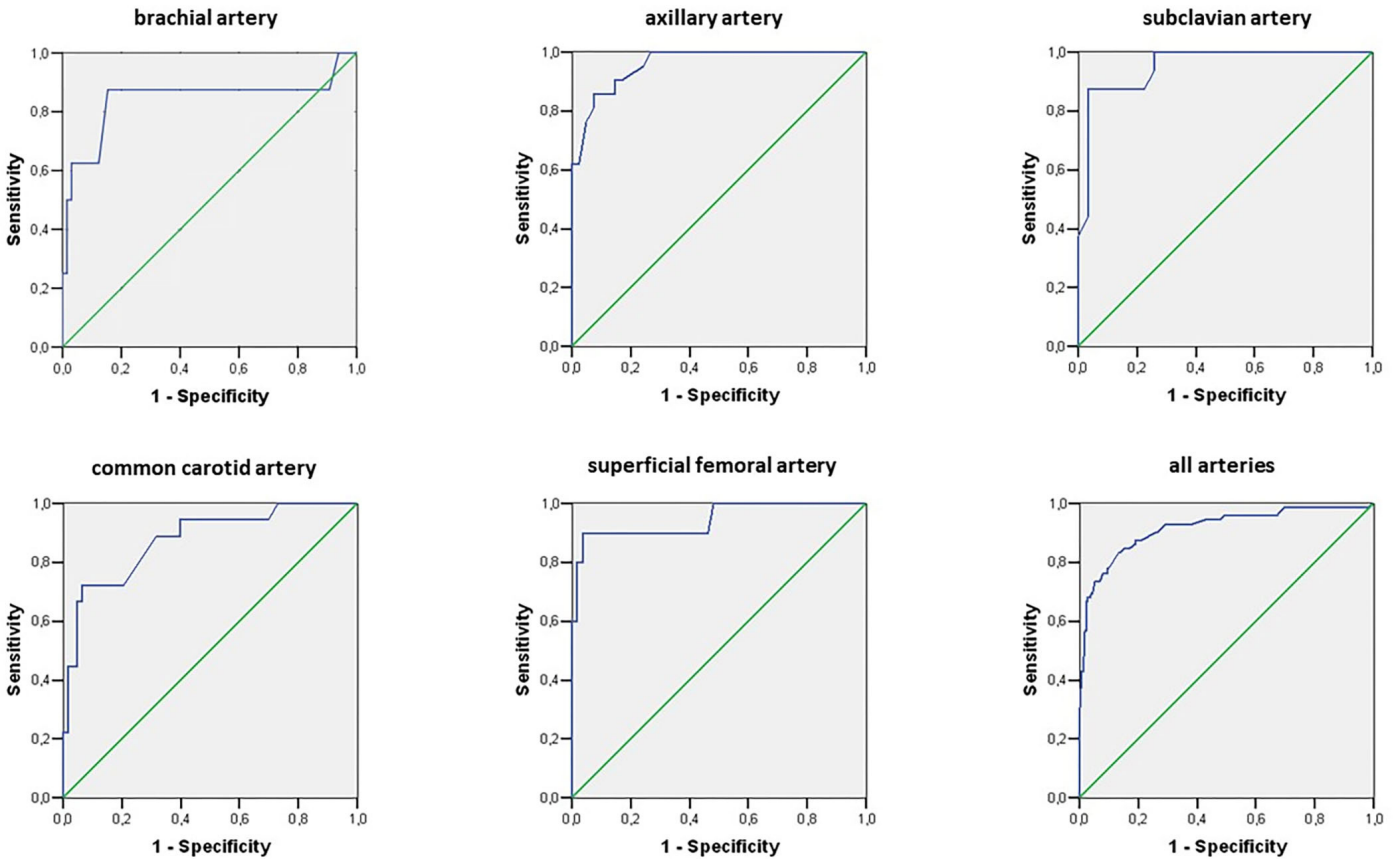
Receiver operating characteristic (ROC) curves for cut-off values of intima-media thickness (IMT) depicting vasculitis in patients with giant cell arteritis. The maximal IMT value from bilateral color duplex ultrasonography measurements was chosen.

Receiver operating characteristic (ROC) curves were calculated (Figure 2). Minimal difference between sensitivity and 1- specificity was chosen for optimal IMT cut-off values for vasculitis. Mann-Whitney and χ^2^ Pearson tests were used to compare between groups. Intrarater reliability was calculated from repeated measurements in 21 vessels of 5 patients and controls. For interrater reliability, 51 IMT measurements in 10 patients and controls were additionally performed by JF. Both readers underwent similar training and used the same equipment. Concordance correlation coefficients for intrarater and interrater reliability were calculated. The *p* < 0.05 were considered to be significant. Statistical analysis was performed using STATA software (version 12.0; StataCorp).

## Results

### Study population

Giant cell arteritis was diagnosed in 81 individuals (TAB was positive for GCA in 31/44 biopsied patients), PMR in 131, and isolated PMR (without concomitant GCA) in 50 patients. Aortitis was diagnosed in 39 out of 126 patients based on CT. Extracranial LV-GCA was diagnosed in 43 patients. In the remaining 83 patients, another diagnosis was confirmed, and they served as non-GCA/PMR controls. Study population characteristics are included in Table 1. Notably, 5 patients with GCA failed to follow up. In the remaining, the diagnosis was sustained. None of the patients with non-GCA were reclassified to GCA. Patients with GCA were significantly older by a mean of 8 years compared with the controls (73 ± 9 vs. 65 ± 10 years). Calcified atherosclerotic plaques covering over 25% of the vessel lumen were significantly less common in isolated PMR compared to GCA and controls. Other patients' characteristics potentially confounding IMT were similar (Table 2). The number of patients referred by different medical specialists is presented in Supplementary Table 1.

**Table 1:** Subtypes of GCA and PMR and structure of controls characterizing 214 included patients.

**Subtypes of GCA and PMR**	***N*** **(%)**	**Structure of non-GCA/PMR controls** **(*N* = 83)**	***N*** **(%)**
GCA	81	Rheumatoid arthritis	26 (31)
Extracranial LV-GCA^*^	43 (53)	Osteoarthritis	12 (14)
Aortitis	39 (48)^**^	Infections	7 (8)
Axillary arteritis	23 (28)	Neoplasms	6 (7)
Subclavian arteritis	18 (22)	Atherosclerosis with related complications	5 (6)
Superficial femoral arteritis	11 (14)	Migraine	4 (5)
Brachial arteritis	8 (10)^***^	Fibromyalgia	3 (4)
PMR	131	Vasculitis (other than GCA)	3 (4)
Isolated PMR^****^	50	Systemic lupus erythematosus	3 (4)
		Spondyloarthropathies	2 (2)
		Neuralgias	2 (2)
		Others	10 (12)

* At least unilateral vasculitis in the large vessel was required to classify the case as GCA,

** based on CT performed in 126 patients,

*** all but one spreading per continuum from axillary arteritis, and

**** without concomitant GCA. GCA, giant cell arteritis; PMR, polymyalgia rheumatica.

**Table 2:** The characteristics of patients.

	**GCA (*N* = 81)**	**Isolated PMR (*N* = 50)**	**Non-GCA/PMR controls (*N* = 83)**
Female/male	53 (65%)/28 (35%)	37 (74%)/13 (26%)	54 (65%)/29 (35%)
Age (years, mean ± SD; min-max)	73^*^± 9; 55–95	69 ± 9; 52–87	65 ± 10; 44–89
Hypertension	53 (65%)	31 (62%)	44 (53%)
Smoking	36 (44%)	14 (28%)	29 (35%)
Diabetes mellitus	13 (16%)	6 (12%)	15 (18%)
Hypercholesterolemia	25/66 (38%)	12/33 (36%)	19/38 (50%)
Calcified atherosclerotic plaques	34 (42%)	11 (22%)^*^	33 (40%)
Upper limbs claudication	4 (4.9%)	0 (0.0%)	1 (1.2%)

* Significant differences (p < 0.05); GCA, giant cell arteritis; PMR, polymyalgia rheumatica; SD, standard deviation.

### IMT measurements

In controls but not in patients with GCA, mean IMT was influenced by age, gender, hypertension, and smoking. The presence of calcified atherosclerotic plaques was associated with increased IMT in PMR, GCA, and controls (Table 3). In LV-GCA cases, IMT in brachial, axillary, subclavian, femoral, and carotid arteries was significantly higher in both vs. controls and isolated PMR. In isolated cranial GCA, large vessel IMT did not differ vs. controls (Table 4). Left CCA IMT was higher compared to the right side in GCA (0.76 ± 0.28 vs. 0.67 ± 0.26; *p* = 0.011) with no significant left to right differences in other arteries in GCA, PMR, and in controls. Receiver operating characteristic (ROC) curves for cut-off values of IMT depicting vasculitis are depicted in Figure 2. Cut-off values for IMT depicting vasculitis vs. controls are listed in Table 5.

**Table 3:** Factors potentially influencing intima-media thickness in GCA, isolated PMR, and controls.

	**GCA (*****N*** = **81)**			**Isolated PMR (*****N*** = **50)**			**Non-GCA/PMR controls (*****N*** = **83)**		
	**Mean IMT ±SD (mm)**	***p***	***R***	**Mean IMT ±SD (mm)**	***p***	***R***	**Mean IMT ±SD (mm)**	***p***	***R***
Female	0.73 ± 0.41	0.375	0.08	0.55 ± 0.20	0.009^*^	0.16	0.49 ± 0.15	< 0.0005^*^	0.28
Male	0.80 ± 0.60			0.62 ± 0.21			0.61 ± 0.19		
Age < 68 years	0.74 ± 0.43	0.505	0.03	0.53 ± 0.17	0.035^*^	0.11	0.50 ± 0.16	< 0.0005^*^	0.35
Age ≥ 68 years	0.77 ± 0.52			0.59 ± 0.22			0.59 ± 0.17		
Hypertension	0.75 ± 0.48	0.675	0.03	0.56 ± 0.20	0.536	0.04	0.56 ± 0.18	0.001^*^	0.19
No hypertension	0.77 ± 0.50			0.58 ± 0.21			0.50 ± 0.16		
Smoking	0.79 ± 0.56	0.288	0.08	0.59 ± 0.19	0.171	0.08	0.58 ± 0.20	0.017^*^	0.13
No smoking	0.73 ± 0.42			0.56 ± 0.21			0.52 ± 0.16		
Diabetes mellitus	0.75 ± 0.68	0.932	0.05	0.65 ± 0.30	0.089	0.10	0.54 ± 0.16	0.570	0.02
No diabetes	0.76 ± 0.45			0.56 ± 0.19			0.53 ± 0.18		
Hypercholesterolemia^**^	0.81 ± 0.59	0.035^*^	0.13	0.65 ± 0.22	0.009^*^	0.15	0.54 ± 0.15	0.854	0.03
No hypercholesterolemia	0.71 ± 0.40			0.56 ± 0.21			0.55 ± 0.17		
Calcified atherosclerotic plaques^***^	0.82 ± 0.58	0.015^*^	0.15	0.68 ± 0.27	< 0.0005^*^	0.20	0.62 ± 0.20	< 0.0005^*^	0.37
No calcified atherosclerotic plaques	0.72 ± 0.42			0.53 ± 0.16			0.48 ± 0.13		

IMT was calculated from measurements in all arteries (axillary, subclavian, superficial femoral, common carotid, and brachial),

* p < 0.05,

** assessment limited to 99 patients and 33 controls, and

*** calcified plaques covering >25% of the vessel lumen. GCA, giant cell arteritis; PMR, polymyalgia rheumatica; SD, standard deviation; R, Spearman's Rank Correlation Coefficient.

**Table 4:** Mean IMT in different affected large arteries in LV-GCA vs. cranial GCA with no LV-GCA, isolated PMR, and controls.

**Artery**	**LV-GCA mean IMT ±SD, mm (#)**	**Cranial GCA with no LV-GCA mean IMT ±SD, mm (*N* = 38)**	**Isolated PMR mean IMT ±SD, mm** **(*N* = 50)**	**Non-GCA/PMR controls mean IMT ±SD, mm (*N* = 83)**
Brachial	0.74^*^± 0.33 (8)	0.40 ± 0.11	0.39 ± 0.13	0.37 ± 0.10
Axillary	1.42^*^± 0.58 (23)	0.55 ± 0.15	0.53 ± 0.19	0.51 ± 0.15
Subclavian	1.31^*^± 0.51 (18)	0.50 ± 0.11	0.50 ± 0.14	0.49 ± 0.15
Common carotid	1.13^*^± 0.43 (24)	0.68 ± 0.17	0.61 ± 0.17	0.57 ± 0.18
Superficial femoral	1.90^*^± 1.20 (11)	0.54 ± 0.17	0.54 ± 0.23	0.50 ± 0.15

^#^Number of patients with vasculitis. Significant findings are marked with asterisks:

* p < 0.05 vs. all other groups. IMT, intima-media thickness; LV-GCA, large vessel giant cell arteritis; PMR, polymyalgia rheumatica; SD, standard deviation.

**Table 5:** Cut-off values for IMT depicting vasculitis in patients with GCA vs. controls.

**Artery**	**AUC**	**Optimal IMT cut-off**			**IMT cut-off for 100% specificity**		
		**to diagnose GCA**			**to diagnose GCA**		
		**IMT (mm)**	**Sens. (%)**	**Spec. (%)**	**IMT (mm)**	**Sens. (%)**	**Spec. (%)**
Brachial	0.842	0.48	88	85	0.96	25	100
Axillary	0.969	0.81	87	94	1.06	62	100
Subclavian	0.974	0.66	100	84	1.35	38	100
Common carotid	0.910	0.73	92	79	1.27	22	100
Superficial femoral	0.958	0.92	91	97	1.55	60	100

The maximal IMT value from bilateral CDU measurements was chosen. AUC, area under the curve; sens., sensitivity; spec., specificity; GCA, giant cell arteritis.

The concordance correlation coefficient for intrarater reliability was 0.96 (95% CI 0.93–0.99), for interrater reliability was 0.96 (95% CI 0.93–0.98), and concordance for the GCA diagnosis was 100%.

## Discussion

The diagnostics of large vessel GCA was improved by introducing new imaging techniques (7, 17–19). However, recommendations for extracranial arteries imaging have a much lower level of evidence compared with cranial arteries (2). IMT cut-off values were not used by the OMERACT group as a modality to define halo signs consistent with vasculitis due to lacking data (11). Defining ultrasound reference ranges could further improve the reproducibility and feasibility of this method.

Among arteries tested in our study, axillary and subclavian arteritis provided the best AUC for the diagnosis of GCA (Table 5), while CCA and axillary were most commonly involved (Table 4). It confirms previous experts' opinions (2, 6, 7) suggesting choosing the axillary artery for LV-GCA assessment. We demonstrated that CCA is the most commonly involved in GCA—a phenomenon that was proved in PET-CT studies but was hard to demonstrate in previous US studies probably due to a problem of differentiation with atherosclerosis that is frequent in this location (20). Axillary artery also demonstrated the lowest difference between highly specific and optimal cut-off values, a phenomenon possibly explained by a lower influence of atherosclerosis in axillary artery compared to arteries prone to develop atherosclerosis (carotid and femoral). However, we confirmed previous observations (21) that expanding the number of arteries examined may add to the diagnosis. In our study, this was the case with CCA (higher number of vasculitis in CCA vs. axillary arteries) and brachial arteries (one case of untypical brachial artery involvement without axillary arteritis). In addition, assessing calcified atherosclerotic plaques in typical places supplies clinically practical information on the potential confounding of IMT results by atherosclerosis (mean IMT was higher in patients with calcified atherosclerotic plaques), which may reduce false positive GCA rates. In our large real-life group, IMT highly specific for vasculitis was higher compared to the previously proposed IMT cut-off values (7, 22). It may be explained by a large cohort and a substantial number of controls with atherosclerosis (patients with atherosclerosis predisposing conditions such as RA were included in the control group and a significant number of patients were referred by neurologists typically with atherosclerosis-related stroke to exclude its vasculitis etiology).

We demonstrated that IMT values may discriminate between GCA and its mimics (Table 4); however, false positive ultrasound results can be found in several diseases (23). An ultrasonographer should consider not only IMT but also the location of the pathological change (11), its structure, and echogenicity, as well as the grade of general atherosclerosis that all add to the final diagnosis of LV-GCA. Importantly, applying cut-off values requires prior identification of atherosclerosis by an ultrasonographer. The methodological obligation in our study was the measurement in a place without atherosclerotic plaque. Interpretation of IMT significance might be enriched by considering the presence of generalized atherosclerosis. The utility of IMT to diagnose or exclude GCA may be low according to the applied cut-off value. Therefore, we proposed two different IMT cut-off values, one based on optimal sensitivity and specificity and the other one that provided 100% specificity in our group, however, with reduced sensitivity. With that approach, lower cut-off values provide a probability for diagnosis instead of a definite diagnosis and necessitate additional ultrasonographic features and a more watchful approach to diagnosing GCA.

Our results indicate that IMT cut-off should be used cautiously to diagnose vasculitis, especially in the case of the presence of atherosclerosis and risk factors of atherosclerosis. Although calcified atherosclerotic plaques were assessed in arterial bifurcations typical for atherosclerosis (to check for the presence of general atherosclerosis) and not at the site of IMT measurement, their presence was correlated with increased IMT both in patients with GCA and controls. Male sex, age ≥ 68, hypertension, and smoking were associated with increased IMT in controls. Of note, these conditions (except for calcified atherosclerotic plaques) did not significantly influence the increase of IMT in patients with GCA suggesting that primary IMT increase by vasculitis might have overcome traditional CV risk factors.

Significantly increased IMT of the left CCA in GCA compared with the right in our observation seems to be only explained by anatomical asymmetry (the left CCA emerges directly from the aortic arch, whereas the right from the brachiocephalic trunk). The difference in left-to-right involvement in LV-GCA has been previously encountered but is not highlighted phenomenon (20, 24, 25). An ultrasonographer may profit from that knowledge when considering CCA arteritis. Interestingly, our brief review of the Medline database performed in April 2022 using a combination of Takayasu, GCA, vasculitis, and “left common carotid artery” or “right common carotid artery” revealed more articles concerning left than right side vascular complications.

An additional secondary result showed a decreased number of calcified atherosclerotic plaques in patients with isolated PMR vs. controls (Table 2). It is interesting in the context of occasionally reported favorable CV outcomes in patients with PMR/GCA (26). However, it requires further studies as some studies reported increased subclinical vascular changes in PMR, even in the absence of vasculitis (27).

Limitations of this study were that non-GCA/PMR controls were younger vs. GCA. However, age did not influence IMT in patients with GCA. The general limitation of the CDU method is that the assessment of IMT remains observer dependent as the measurement in a place without atherosclerotic plaques requires prior categorization into atherosclerosis/non-atherosclerosis. The possible limitation of the ultrasound-based study is that the observer might be reluctant to classify CCA with slightly increased IMT as vasculitis because this artery is commonly involved in atherosclerosis (13). This location might be an even more common site of arteritis in GCA, as demonstrated by PET examinations (20), but it should be examined by ultrasound with caution to conclude on vasculitis at least until more studies on CCA vasculitis vs. atherosclerosis will be available. We believe that determining the grade of atherosclerosis by assessing the presence of typical plaques in the carotid bulb and femoral arteries is feasible, to gain more information on the nature of the increase of IMT in CCA. The limitation of the study includes a lack of data on patients' obesity.

The strengths of the study include real-life referrals, examination of multiple large arteries, which, to the best of our knowledge, were not vastly analyzed previously, thorough analysis of potential confounders, and excellent interrater reliability.

In conclusion, we proposed IMT cut-off values to assess the probability of the diagnosis of GCA. Applying cut-off values may be enriched by prior identification of confounding by atherosclerosis, age, gender, hypertension, and smoking. They performed best in axillary and subclavian arteritis but examination of the other arteries may add to the diagnosis of GCA.

## Data availability statement

The raw data supporting the conclusions of this article will be made available by the authors, without undue reservation.

## Ethics statement

The studies involving human participants were reviewed and approved by Ethics Committee of Pomeranian Medical University. The patients/participants provided their written informed consent to participate in this study.

## Author contributions

MM and JF performed the examinations and organized the database. MM wrote the manuscript. All authors contributed to the conception, design, analysis interpretation of the study, and manuscript revision, as well as read and approved the submitted version.
