# Supplementary material for: Intima-media thickness cut-off values depicting “halo sign” and potential confounder analysis for the best diagnosis of large vessel giant cell arteritis by ultrasonography
Source: Front Med (Lausanne). 2022 Dec 13;9:1055524. doi: 10.3389/fmed.2022.1055524 (PMC9792608; doi:10.3389/fmed.2022.1055524)
Supplement: Supplementary file 1 [file Table_1.docx]

**SUPPLEMENTARY MATERIAL**

The Supplementary Material for this article can be found online

Table 1s. Number of patients referred by different medical specialists (N=214)

| Referral by | Number of patients referred |
| --- | --- |
| ophthalmologists | 43 (20%) |
| internists (with subspecialities) | 41 (19%) |
| rheumatologists | 34 (16%) |
| neurologists | 18 (8%) |
| infectious medicine specialists | 9 (4%) |
| angiologists | 7 (3%) |
| the others | 6 (3%) |
| family doctors | 0 (0%) |
